# The effect of ethical leadership on service recovery performance: A moderated mediation model of organizational virtuousness and trait mindfulness

**DOI:** 10.3389/fpsyg.2022.1036099

**Published:** 2022-11-29

**Authors:** Ding Ma, Nauman Wajid, Muhammad Khalid Anser, Zafar-Uz-Zaman Anjum, Xiaoyun Jia

**Affiliations:** ^1^Graduate Institute for Taiwan Studies, Xiamen University, Xiamen, Fujian Province, China; ^2^NUST Business School, National University of Sciences and Technology, Islamabad, Pakistan; ^3^Faculty of Business and Management Sciences, The Superior University, Lahore, Pakistan; ^4^Department of Management Sciences, COMSATS University Islamabad, Lahore Campus, Lahore, Punjab, Pakistan; ^5^Business School, Foshan University, Foshan, China; ^6^School of Natural and Computational Sciences, College of Sciences, Massey University, Auckland, New Zealand

**Keywords:** ethical leadership, organizational virtuousness, service recovery performance, trait mindfulness, frontline employees

## Abstract

In the present study, we first examined the relationship between ethical leadership and frontline employees’ (FLEs’) service recovery performance (SRP) and then tested the mediating role of organizational virtuousness in the relationship between ethical leadership and SRP in service contexts. Finally, we examined the moderating effect of FLE trait mindfulness on the direct relationship between ethical leadership and organizational virtuousness, as well as the indirect relationship between ethical leadership and SRP, *via* organizational virtuousness. Three-waved survey data collected from 273 supervisor-employee dyads in different service sector organizations supported our hypothesized relationships. In addition to important theoretical implications, the study carries useful practical implications, particularly for managers who are concerned about improving SRP in the service contexts.

## Introduction

Service sector firms are more prone to service failures due to consistently increasing customers’ demands and the heterogeneous nature of services (Ali et al., 2020; Yoon, 2022). Thus, the role of service recovery performance (SRP), defined as employees’ effectiveness to address customers’ complaints (Boshoff and Allen, 2000) become more important in services contexts (Jerger and Wirtz, 2017). A few studies have shown that leadership positively influences SRP (Tuan and Thao, 2018; Luo et al., 2019). Yet, the role of ethical leadership in SRP has not been studied. This omission is critical because effective leadership is one of the key antecedents of employees’ work-related outcomes and plays a dominant role in helping organizations to respond to challenging situations (Tuan and Thao, 2018), such as service failures. We address this gap by proposing a model that explains why and when ethical leadership is linked with frontline service sector employees’ (FLEs) SRP. We use social learning theory (Bandura, 1977, 1986) to theorize the proposed model between ethical leadership and SRP.

Ethical leadership is defined as *“the demonstration of normatively appropriate conduct through personal actions and interpersonal relationships, and the promotion of such conduct to followers”* (Brown et al., 2005, p: 120). Ethical leadership has been considered in this work, as ethical leaders emphasize on followers’ professional and personal development, and thus goes beyond the contractual obligations (Yidong and Xinxin, 2013; Tseng and Wu, 2017; Ali et al., 2022a). Ethical leadership ensures that all the rights of an organization’s stakeholders, including customers are protected and their voices and concerns are heard (Brown et al., 2005). As such, drawing on social learning theory, we argue that FLEs working under ethical leadership would imitate ethical leader behaviors and listen to customers’ concerns related to service quality and address the raised concerns. Thus, we understand that ethical leadership has theoretical relevance with SRP.

Proceeding further, to enhance our understanding as to why ethical leadership positively influences SRP, we propose that perceived organizational virtuousness mediates the positive association between ethical leadership and SRP. Organizational virtuousness refers to employees’ perceptions that various virtues, such as forgiveness, compassion, and integrity are practiced and valued in the organization (Cameron and McNaughtan, 2014). We focus on organizational virtuousness because based on social learning theory, we understand that ethical leaders’ demonstration of honesty, integrity, and high moral standards, trustworthiness, empathy, and concern for others are likely to be learned and imitated by followers. We further argue that consistent exhibition of such behaviors in the organization can positively influence employees’ perceptions of organizational virtuousness that can enable them to deal with consumers’ diverse complaints.

**Figure 1:**
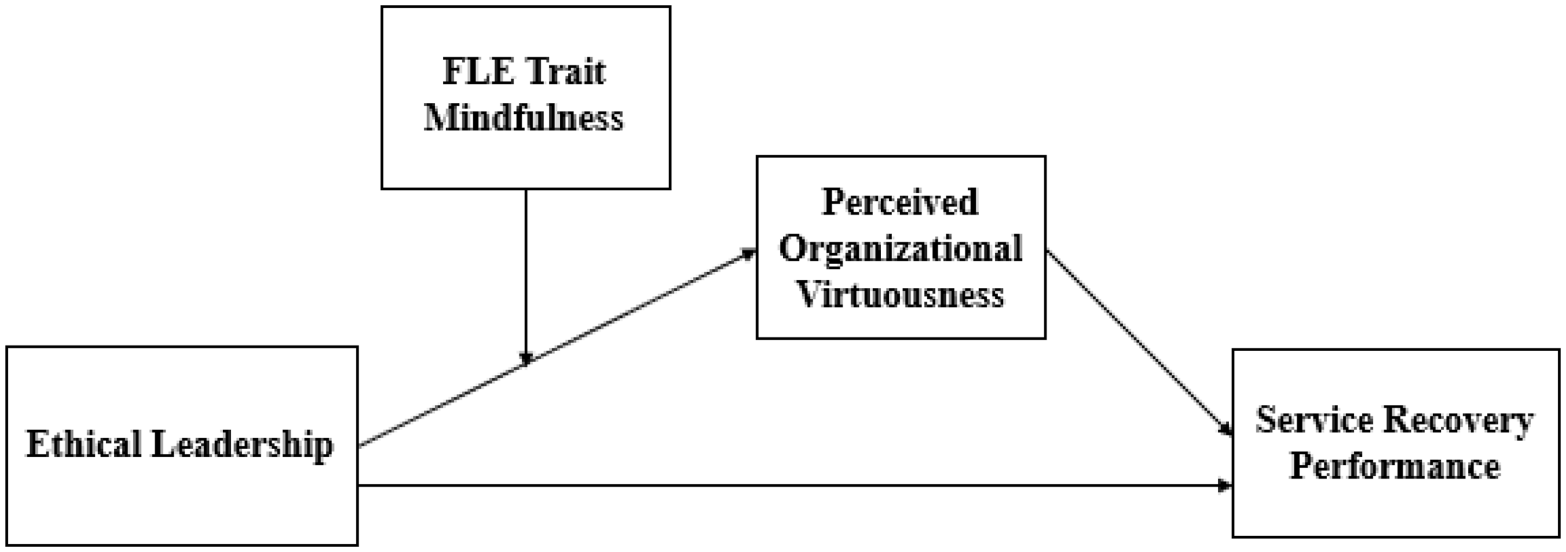
The proposed model.

Finally, we theorize that the extent to which ethical leadership impacts organizational virtuousness is contingent on employee trait mindfulness – the level of individuals’ being conscious of their thoughts and feelings (Ryan and Brown, 2003). We argue that FLEs high on mindfulness are likely to pay attention to and learn from ethical leaders. Thus, we understand that FLEs high on trait mindfulness can have stronger perceptions of organizational virtuousness and benefit more from ethical leadership in terms of performing SRP. The proposed model is presented in Figure 1.

The present study contributes and enhances the networks of the antecedents and outcomes of SRP and ethical leadership, respectively. Another key contribution is to the organizational virtuousness literature (Eisenberger et al., 2001). We advance the literature on organizational virtuousness by showing that it mediates the ethical leadership-SRP link. Finally, we advance the service literature on mindfulness (e.g., Anasori et al., 2020; Gip et al., 2022) by demonstrating that the positive effects of ethical leadership on SRP, *via* organizational virtuousness is strengthened when trait mindfulness is high.

## Hypotheses development

### Ethical leadership and FLE service recovery performance

The literature (e.g., Ferdig, 2007; Kalshoven et al., 2011; Abdullah et al., 2019; Khan et al., 2019) suggests that ethical leaders focus on the rights of different stakeholders including employees, organizations, society, and customers. Moreover, given their focus on protecting other stakeholders’ rights, ethical leaders listen to others’ concerns that helps them understand others’ perspectives (Brown et al., 2005), thereby addressing others’ concerns more effectively. According to social learning theory, employees are likely to emulate such a focus of ethical leadership on the rights of different stakeholders, including customers. As such, employees working under ethical leadership may listen to and address customers’ complaints effectively.

Additionally, fairness and honesty are among the key features of ethical leadership (Brown and Treviño, 2006; Resick et al., 2011) and ethical leaders treat followers fairly and honestly. Drawing from social learning theory, we argue that employees of ethical leadership treat their customers’ complaints fairly and honestly. Indeed, leaders’ honesty is the foundation from which they can win followers’ trust (Piccolo et al., 2010) and inspire them to exert extra efforts to perform their work roles (Ali et al., 2022a). Importantly, ethical leaders serve as a role model for employees to help them make an honest use of organizational time and resources (e.g., Zhu et al., 2004; Eisenbeiß and Brodbeck, 2014) to handle customers’ diverse complaints. Thus, the following hypothesis.

*Hypothesis 1:* Ethical leadership is positively related to SRP.

### Organizational virtuousness as a mediator

Ethical leadership demonstrates concern, kindness, respect, forgiveness, high moral standards, integrity, and compassion through their behaviors and actions (Brown et al., 2005). Ethical leaders also recognize and appreciate followers’ efforts, listen to them responsively and take care of their personal and professional development (Brown and Treviño, 2006; Anser et al., 2021a). Social learning theory suggests that followers observe and imitate their leaders. Thus, drawing on social learning theory, we argue that FLEs working under ethical leadership will receive and demonstrate moral values, a sense of self transcendence, compassion, kindness, respect, forgiveness, and concern for others, such as peers through their behaviors and actions. We understand that leaders’ and employees’ consistent demonstration of such virtues is likely to shape employees’ perceptions of organizational virtuousness.

Proceeding further, employees’ perceptions of organizational virtuousness foster feelings of gratitude in respect of the work settings (Tsachouridi and Nikandrou, 2016), and thus employees may feel obliged to adapt their behaviors and actions to benefit the organization and its customers (Eisenberger et al., 2001). Scholars have highlighted that optimism, a key aspect of organizational virtuousness improves employees’ confidence in their abilities (Rego et al., 2015; Magnier-Watanabe et al., 2020), and thus can enhance their ability to deal with service failures more effectively. Further, compassion and interpersonal trust provide employees with a sense of autonomy and mutual cooperation among employees (Hur et al., 2017). Interpersonal relations and mutual cooperation enable employees to address customers’ complaints more effectively. Together, we propose the following hypothesis.

*Hypothesis 2:* Employee perceived organizational virtuousness mediates the positive association between ethical leadership and SRP.

### The moderating role of FLE trait mindfulness

Social learning theory suggests that followers’ learning from and imitation of their leaders vary across individuals. Trait mindfulness emphasizes attention and awareness (Ryan and Brown, 2003) and therefore, we argue that it enables FLEs to be more aware of and attentive to ethical leaders’ behaviors and can develop an enhanced sense of organizational virtuousness. We suggest employees more attentive to ethical leadership behaviors are better able to focus their attention on present experiences (Anasori et al., 2020; Loureiro et al., 2020). Consequently, FLEs high trait mindfulness demonstrate better ability to imitate ethical leader behaviors and therefore, can exhibit more compassion and care for their peers through their behaviors. As such, compared to others, FLEs high on trait mindfulness are more likely to adopt and demonstrate virtues that comprise organizational virtuousness. Thus, it is expected that the influence of ethical leadership on FLEs’ perceived organizational virtuousness will be stronger for FLEs high (vs. low) on trait mindfulness.

*H3:* FLE trait mindfulness moderates the positive relationship between ethical leadership and perceived organizational virtuousness, such that this relationship is stronger when trait mindfulness is high (vs. low).

The present study theorized earlier informed by social learning theory that ethical leadership enhances perceived organizational virtuousness, which in turn positively influences SRP. Additionally, as proposed above (H3), the influence of ethical leadership on perceived organizational virtuousness is stronger when the level of FLE trait mindfulness is high (vs. low). Therefore, by combining the logic from H2 and H3, we argue that it is likely that the indirect relationship between ethical leadership and SRP, *via* organizational virtuousness is stronger when trait mindfulness is high (vs. low). Thus, the following hypothesis is proposed.

*H4:* FLE trait mindfulness moderates the indirect (via RBSE) relationship between inclusive leadership and SRP, such that the relationship is stronger when employee trait mindfulness is high (vs. low).

## Materials and methods

### Data collection and analysis

A three-wave (separated by a two-month lag) survey data was collected from 273 employees working in 60 service sector firms in China. These firms were selected because of easy access to these firms that was managed using personal and professional contacts. We received the list of FLEs of each firm and randomly chose 500 FLEs, who were provided with a cover letter containing a promise of confidentiality and general purpose of the study. Consent to participate in data collection was received from 356 FLEs.

We received 325 and 309 responses in the first and second waves, respectively. In the first, wave, we collected data about ethical leadership, the moderator (FLE trait mindfulness) and demographic variables. In the second wave, data about perceived organizational virtuousness was collected from FLEs. Data from two waves were matched using unique codes. In the third wave, supervisor rated FLEs’ SRP. In total, we received 288 supervisors’ responses. After screening the data for attention checks and missing values, 273 matched responses were retained and used for hypotheses testing.

The final sample consisted of 56.4% males and 43.6% females. Of these, 23.1% had completed 10 years of schooling, 20.9% had 12 years of education, 27.8% had undergraduate degree, and 28.2% had a master’s degree or above. Data were analyzed using structural equation modeling in Mplus (8.6).

Data were collected from the sample in three rounds using self-administered survey technique to reduce the common method bias. The two-month lag allows sufficient time to overcome common method bias. Two-source data also mitigates common method bias.

### Measures and variables

Unless otherwise stated, the items that measured all of the variables in this study used a 5-point scale, from 1 (strongly disagree) to 5 (strongly agree). All of the items were coded such that high scores equated with the higher levels of the constructs. All the constructs and items are presented in Table 1.

**Table 1:** Means and correlations.

	Mean	SD	1	2	3	4	5	6	7
1. Ethical leadership	3.48	1.01							
2. Organizational virtuousness	3.57	1.03	0.35^**^						
3. Service recovery performance	3.03	1.08	0.39^**^	0.46^**^					
4. Trait mindfulness	3.45	0.98	0.06	0.22^**^	0.00				
5. Age	32.05	5.1	−0.08	0.00	−0.13^*^	−0.03			
6. Gender			−0.06	0.00	0.00	0.05	0.07		
7. Education			−0.01	0.04	−0.05	0.03	−0.05	0.03	
8. Tenure	3.11	1.45	−0.06	−0.03	0.03	−0.03	0.11	0.01	−0.14^*^

*p  < 0.05 and **p < 0.01. Sample size (N) = 273.

#### Ethical leadership

We measured ethical leadership by adopting a ten-item scale (α = 0.93) from Brown et al. (2005). Sample item: “My supervisor listens to what employees have to say.”

#### Organizational virtuousness

Perceived organizational virtuousness was measured using a 15-item scale (Cronbach Alpha = 0.91) by Cameron et al. (2004). Sample item: *“We have very high standards of performance, yet we forgive mistakes when they are acknowledged and corrected.”*

#### SRP

FLEs’ SRP was assessed by adapting a four-item scale (*α = 0.88*) from Kim et al. (2009). Sample item: *“I am pleased with the manner in which this employee resolved the service failure.”*

#### Trait mindfulness

FLE Trait mindfulness was assessed by using 6 items *(α = 0.94)* from Ryan and Brown (2003). The items were measured on a five-point scale (1 = almost always, and 5 = almost never), with higher scores reflecting greater trait mindfulness. Sample item: *“I find it difficult to stay focused on what’s happening in the present.”*

#### Control variables

Employees may differ in a number of ways and differences in such areas as age, gender, tenure with the organization, and education can confound the results. Thus, we controlled for age, gender, education, and tenure with the organization.

## Results

Means and correlations for the variables of the study are presented in Table 1.

### Measurement model

We used confirmatory factor analysis (CFA) to evaluate the measurement model, which consisted of ethical leadership, perceived organizational virtuousness, SRP, and trait mindfulness. All the items loaded significantly on their respective constructs. The fit indices, χ^2^ (554) = 1646.19, χ^2^/df = 2.97, IFI = 0.90, TLI = 0.90 and CFI = 0.90 and RMSEA = 0.07 show that the measurement model has a good fit with the data.

Maximum variance shared (MSV), average variance extracted (AVE), and average shared variance (ASV) of all the variables are presented in Table 2. The scales also demonstrated a satisfactory level of discriminant validity with ASV and MSV < AVE (Table 3). The square roots of AVE (Table 2) for each variable in the study were greater than their inter-construct correlations. Thus, the scales also demonstrated convergent validity.

**Table 2:** Discriminant validity and convergent validity.

Construct	1	2	3	4	AVE	MSV	ASV
1. Ethical leadership	**0.79**				0.63	0.18	0.10
2. Organizational virtuousness	0.36	**0.76**			0.58	0.24	0.14
3. SRP	0.43	0.49	**0.79**		0.62	0.24	0.14
4. Trait mindfulness	0.04	0.23	−0.03	**0.77**	0.60	0.05	0.02

*N* = 273. AVE = Average variance extracted. MSV = Maximum shared variance. ASV = Average shared variance. Bolded values on the diagonals of columns 2 to 5 are the square root values of AVE. SRP = Service recovery performance.

**Table 3:** Hypotheses results.

Total effect	B	SE
Ethical leadership → SRP	0.42^**^	0.06
Direct paths		
Ethical leadership → SRP	0.28^**^	0.06
Ethical leadership → Organizational virtuousness	0.36^**^	0.06
Organizational virtuousness → SRP	0.39^**^	0.06
Indirect paths		
Ethical leadership → Organizational virtuousness → SRP	0.14^**^	0.03
Moderated paths		
Ethical leadership*Trait mindfulness → Organizational virtuousness	0.20^**^	0.05
Ethical leadership*Trait mindfulness → Organizational virtuousness → SRP	0.08^**^	0.03

**p  < 0.01. Sample size (N) = 273 (bootstrapping by specifying a sample of size 5,000). SRP = Service recovery performance.

### Hypotheses testing

As shown in Table 3, ethical leadership was positively related to SRP (*B* = 0.42, *SE* = 0.06, *p* < 0.01). Thus, hypothesis 1 was supported. Furthermore, the indirect relationship (*via* perceived organizational virtuousness) between ethical leadership and SRP was significant (*B* = .14 s, *SE* = 0.03, *p* < 0.01), showing that perceived organizational virtuousness mediates the positive relationship between ethical leadership and SRP. Thus, hypothesis 2 was also supported.

**Figure 2:**
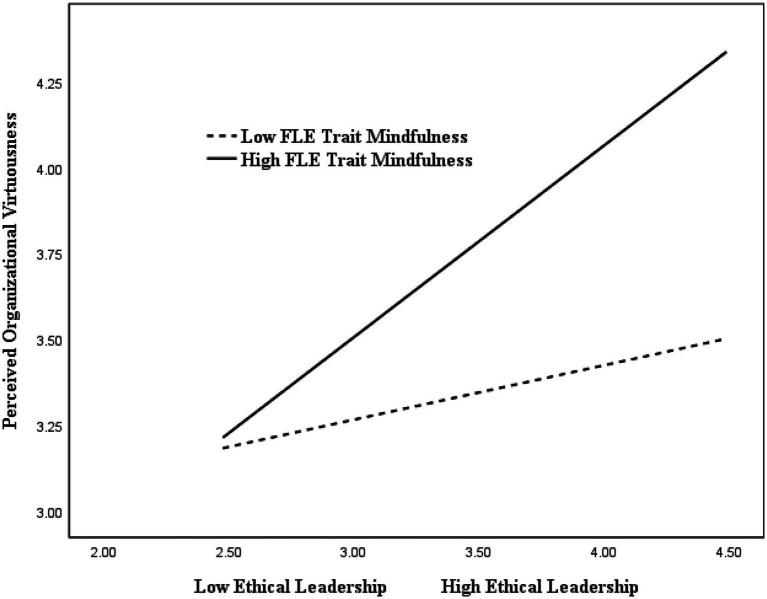
FLE trait mindfulness as a moderator of the relationship between ethical leadership and perceived organizational virtuousness.

Hypotheses 3 and 4 were tested by adding the interaction term of FLE trait mindfulness and ethical leadership to the indirect effects model. The moderation results (Table 3) show that the effect of the interaction between FLE trait mindfulness and ethical leadership on perceived organizational virtuousness was significant (*B = 0.20, SE = 0.05, p < 0.01*). The precise nature of this moderated path is depicted in Figure 2. Simple slope plots are showing two conditional values of this relationship between ethical leadership and perceived organizational virtuousness at two different levels of the moderator: low trait mindfulness (i.e., one standard deviation below the mean) and high trait mindfulness (i.e., one standard deviation above the mean). The association between ethical leadership and perceived organizational virtuousness was significant (*B = 0.56, SE = 0.07, p < 0.01*) when trait mindfulness was high, while the relationship was insignificant (*B = 0.16, SE = 0.07, ns*) when trait mindfulness was low. Thus, hypothesis 3 was supported.

Finally, the moderation-mediation analysis illustrated that the conditional indirect association (*via* perceived organizational virtuousness) between ethical leadership and SRP was significant [*B = 0.22, SE = 0.05, CI =* (0.*13, 0.32*] when FLE trait mindfulness was high, while the relationship was insignificant (*B = 0.046, SE = 0.04, ns*) when FLE trait mindfulness was low. Moreover, the index of moderated mediation was significant for the hypothesized indirect (*via* perceived organizational virtuousness) relationship between ethical leadership and SRP [*index = 0.08, SE = 0.03, CI = (0.02, 0.14)*]. Thus, hypothesis 4 was supported.

## Discussion and theoretical contributions

Drawing on social learning theory (Bandura, 1977, 1986), we hypothesized that supervisory ethical leadership is positively related to SRP, both directly and *via* perceived organizational virtuousness. We also proposed that FLE trait mindfulness moderates the relationship between ethical leadership and perceived organizational performance and the indirect relationship between ethical leadership and FLEs’ SRP. Our time-lagged survey data collected from two sources supported our hypotheses.

### Theoretical contributions

Our study makes several contributions in the literature. First, we find evidence of the positive influence of ethical leadership on FLEs’ SRP in the service contexts. Ethical leadership contributes positively to a number of work-related behaviors and attitudes, such as employees’ ethical behavior, organizational commitment, work engagement, psychological wellbeing, good citizenship, affective commitment and job satisfaction (Avey et al., 2012; Chughtai et al., 2015). The literature also indicates that ethical leadership helps employees to unlearn obsolete and destructive behaviors and practices and can positively influence explorative and exploitative learning (Usman and Hameed, 2017; Ali et al., 2022a). However, the literature has not yet explored the influence of ethical leadership on SRP. Thus, our study enhances the networks of antecedents and outcomes of SRP and ethical leadership, respectively.

Second, we contribute to the literature on organizational virtuousness (Searle and Barbuto 2011; Magnier-Watanabe et al., 2020; Sun and Yoon, 2022) by revealing that, in addition to the direct effect, ethical leadership operates through organizational virtuousness to impact SRP. In line with social learning theory, our findings indicate that followers learn from and imitate ethical leaders and demonstrate forgiveness, compassion, integrity, empathy, and other positive characteristics through their behaviors and actions. A consistent demonstration of these behaviors by leaders and followers leads to organizational virtuousness, which in turn improves their SRP. Although studies suggest that leads to several positive outcomes (Searle and Barbuto 2011; Magnier-Watanabe et al., 2020), such as employee performance and job satisfaction, it has received little attention in terms of its influence on SRP and its role in the ethical leadership-FLEs’ SRP. Thus, our study brings to the fore important yet ignored roles of organizational virtuousness and enhance the networks of its antecedents and outcomes.

Finally, the present study integrates an important individual difference – trait mindfulness – into our model. Owing to individual differences, followers may have different levels of attention to ethical leader behaviors. Past research suggests that trait mindfulness negatively influences depression, anxiety, work withdrawals, burnout, and job stress (Good et al., 2016; Zhang et al., 2019) and that it has several positive outcomes, such as emotional regulations and performance (Zhang et al., 2019). The present study is the first to integrate the construct of trait mindfulness in the context of ethical leadership and employee outcomes. Trait mindfulness explains when ethical leadership is more effective in shaping FLEs’ perceptions of organizational virtuousness and facilitating SRP and thus addresses the calls (e.g., Zhang et al., 2019) for more studies on this construct in the work domain.

### Practical implications

First, our study findings provide insights to leaders about how their ethical leadership behaviors can enhance FLEs’ SRP. The findings show that ethical leadership plays a critical role in encouraging employees to take steps to solve customers’ complaints that could potentially result in important positive outcomes for service organizations. Service organizations are therefore encouraged to develop and cultivate ethical leadership behaviors in managers. There are a number of options in this context. This may for example be relied upon through the demonstration of ethical leadership behaviors, such as integrity, compassion, and honesty in addition to the provision of leadership development programs. These programs can make leaders aware of the importance that ethical leadership for employee behavior in improving FLEs’ SRP.

The study findings also point to the importance of focusing on organizational virtuousness as a cultural value within service organizations. Organizational virtuousness points to particular facets of culture that organizations should focus on including self-transcendence, compassion, kindness, respect, forgiveness, and concern for others. We suggest that senior hotel leaders have an important role in shaping the culture of service organizations and they can perform this role in a number of ways. First, senior leaders must be themselves role models in terms of the dimensions of organizational virtuousness to propagate it to others in the organization. Second, where senior leaders communicate through their words and actions virtuous values and behaviors these can contribute to the development of a culture that espouses the values of organizational virtuousness.

Our findings concerning the moderating role of trait mindfulness also have important managerial implications. It suggests that hospitality organizations should pay attention to individual differences in selection, assessment, and development processes. Service organizations could significantly reap the benefits of ethical leadership when they select employees that are high on trait mindfulness. Trait mindfulness seems to hold important promise to strengthen the effects of ethical leadership on followers’ perceptions of organizational virtuousness and SRP. Therefore, it should be systematically measured in selection processes. Trait mindfulness can also be incorporated into the design of training programs.

### Limitations and future research directions

This study is not without limitations. For instance, we focused on service sector firms. It would be interesting to examine the relationships by collecting data from manufacturing sector firms. According to Mason and Leek (2008), what firms learn varies with the context. Thus, testing our hypotheses in different contexts is an important area of future research. Additionally, the relationship between ethical leadership and learning can be intervened and mediated through a number of mediators. For instance, ethical leadership emphasize on a two-way communication that facilitate knowledge sharing and new knowledge creation (Usman and Hameed, 2017) that leads to individual and organizational learning (Usman and Ahmad, 2017; Usman et al., 2019). This suggests knowledge sharing and knowledge creation can mediate the relationship between ethical leadership and learning. Although the inclusion of these variables in our model would have made our model cumbersome, keeping in view the importance of knowledge sharing and knowledge creation in gaining sustained competitive advantage (Mason and Leek, 2008; Chung et al., 2015; Usman et al., 2019; Anser et al., 2021b), we suggest that studying the mediatory role of these variables in the relationship between ethical leadership and learning can offer important theoretical and practical implications.

Moreover, several additional constructs potentially moderate the relationship between ethical leadership and employee SRP. For example, harmonious work passion inspires employees to engage in work activities to derive pleasure and positive experiences that lead them to engage in extra-role efforts (Anser et al., 2021c). It is also likely that employees high on harmonious work passion will benefit more from ethical leadership and thus elevate the effectiveness of ethical leadership for shaping employees’ SRP. Future studies should therefore investigate the role of harmonious work passion to enhance our understanding of the complexities engaged in the leadership-SRP. Finally, other leadership styles such as spiritual leadership (Anser et al., 2021b; Ali et al., 2022b,c) and servant leadership (Usman et al., 2022) can also positively influence SRP. Thus, future studies should also examine the association between other positive leadership styles and SRP to offer a more complete understanding of this link between leadership and SRP.

## Data availability statement

The raw data supporting the conclusions of this article will be made available by the authors, without undue reservation.

## Ethics statement

The studies involving human participants were reviewed and approved by the Ethics Committee of Business School of Foshan University. The patients/participants provided their written informed consent to participate in this study.

## Author contributions

DM, MA, ZZA, NW, and XJ: definition of research objectives, models, and hypotheses and data analysis plan, and final approval. NW, ZZA, and MA: the provision of materials (i.e., questionnaires). MA and XJ: data collection and data analysis. MA, ZZA, DM, and NW: principal article writing. XJ, NW, and DM: article revision and proofreading. All authors contributed to the article and approved the submitted version.

## Conflict of interest

The authors declare that the research was conducted in the absence of any commercial or financial relationships that could be construed as a potential conflict of interest.

## Publisher’s note

All claims expressed in this article are solely those of the authors and do not necessarily represent those of their affiliated organizations, or those of the publisher, the editors and the reviewers. Any product that may be evaluated in this article, or claim that may be made by its manufacturer, is not guaranteed or endorsed by the publisher.
